# International Comparison of Geriatric‐Associated Variables in Major Gastroenterological Surgery Between National Clinical Database and American College of Surgeons National Surgical Quality Improvement Program

**DOI:** 10.1002/ags3.70021

**Published:** 2025-04-21

**Authors:** Yasuhide Kofunato, Xane Peters, Arata Takahashi, Mark E. Cohen, Hiraku Kumamaru, Mitsukazu Gotoh, Yoshihiro Kakeji, Yasuyuki Seto, Yuko Kitagawa, Ken Shirabe, Hideki Ueno, Hiroaki Miyata, Clifford Y. Ko, Shigeru Marubashi

**Affiliations:** ^1^ Department of Hepato‐Biliary‐Pancreatic and Transplant Surgery Fukushima Medical University Fukushima City Fukushima Japan; ^2^ American College of Surgeons National Surgical Quality Improvement Program (NSQIP) Chicago Illinois USA; ^3^ Department of Health Policy and Management School of Medicine, Keio University Tokyo Japan; ^4^ Department of Healthcare Quality Assessment The University of Tokyo Bunkyou‐ku Tokyo Japan; ^5^ Osaka General Medical Center Osaka City Osaka Japan; ^6^ Division of Gastrointestinal Surgery, Department of Surgery Kobe University Graduate School of Medicine Kobe City Hyogo Japan; ^7^ Department of Gastrointestinal Surgery Graduate School of Medicine, the University of Tokyo Bunkyou‐ku Tokyo Japan; ^8^ Department of Surgery Keio University School of Medicine Shinjuku‐ku Tokyo Japan; ^9^ Division of Hepatobiliary and Pancreatic Surgery, Department of General Surgical Science Gunma University Graduate School of Medicine Maebashi City Gunma Japan; ^10^ Department of Surgery National Defense Medical College Tokorozawa City Saitama Japan

**Keywords:** database, gastroenterological surgery, geriatric surgery, international comparison

## Abstract

**Backgrounds:**

Incidence of malignant disease in older patients has been increasing. These geriatric patients have more comorbidities and frailty than younger patients, necessitating different approaches in evaluation and treatment. Geriatric surgery studies in Japan have followed those conducted in the US. The aims of this study were to compare trends in geriatric variables for patients who underwent gastroenterological surgeries between two countries, and to elucidate the characteristics of them.

**Study Design:**

Geriatric variables and outcomes were analyzed via nationwide databases in both countries. Subjects were defined as patients with age ≥ 65 who underwent seven major gastroenterological surgeries for malignant disease. Basic statistical values were compared between them.

**Results:**

A total of 2703 patients in the National Clinical Database (NCD) and 1342 patients in the American College of Surgeons National Surgical Quality Improvement Program (NSQIP) were included. Among preoperative comorbidities, dyspnea, hypertension, bleeding disorder, and emergency cases increased with age in both databases, while the rates of obesity and emergency cases were more frequent in NSQIP. Most postoperative complications were not significantly associated with age in either database. Geriatric‐associated preoperative variables and outcomes varied with age in both countries. Cognitive variables (history of dementia, surrogate‐signed consent, and delirium) were similar between the two databases. However, mobility elements (use of mobility aid, fall history, high fall risk, and new use of mobility aid) and postoperative functional dependency were more frequent in NSQIP than NCD.

**Conclusion:**

Geriatric‐associated variables and outcomes changed similarly with age in both countries, while mobility and function were different between the two.

## Graphical abstract

Geriatric surgical risk factors and outcomes were compared between the US and Japanese databases. Geriatric‐associated preoperative variables and outcomes similarly increased with age. Mobility and functional domains differed between the two databases.

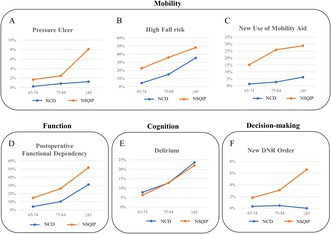

**Abbreviations**
ACS‐NSQIP: American College of Surgeons National Surgical Quality Improvement Program
ASA‐PS: American Society of Anesthesiologists—physical status
DG: distal gastrectomy
Eso: esophagectomy
HEP: hepatectomy
LAR: low anterior resection of the rectum
NCD: National Clinical Database
PD: pancreaticoduodenectomy
RH: right hemicolectomy
TG: total gastrectomy

## 1 Introduction

Life expectancies are continuously increasing, and the proportions of aged people have become higher in both Japan and the US. Malignant diseases are one of the top causes of deaths in both countries, and it is estimated that 50.2%–65.5% of the population in Japan and 37.7%–39.3% in the US experience malignant neoplasms at least once in their lifetime [1, 2]. Surgical care remains the mainstay for curative intent among multidisciplinary treatment and has seen rapid development in patient safety and outcomes due to various devices and surgical innovations. Surgical treatment of malignancy continues to concentrate on the geriatric population and represents a major priority of study in these patients [3].

Geriatric patients tend to have more preoperative comorbidities [4, 5, 6, 7] and frailty [8, 9, 10], reflecting lower physical function and higher need for assistance than younger patients. These vulnerabilities may result in higher morbidity and mortality in surgical treatment than in younger generations [11]. Therefore, geriatric patients require special consideration for evaluation and treatment.

The American College of Surgeons National Surgical Quality Improvement Program (ACS‐NSQIP) started the Geriatric Surgery Pilot Project in the US in 2014, which targeted patients age 65 years or older who underwent the surgical operations of all subspecialties except trauma and transplantation in 21 participating hospitals [12]. One result of this study was the establishment of risk models for geriatric‐specific outcomes (pressure ulcer, delirium, new mobility aid use, and functional decline), based on preoperative geriatric‐associated variables (mobility aid use, history of dementia/cognitive impairment, origin status on admission, hospice/palliative care on admission, fall history, and surrogate‐signed consent). This was incorporated into the web‐based NSQIP Surgical Risk Calculator (https://riskcalculator.facs.org/RiskCalculator/). In collaboration with NSQIP, a geriatric surgery pilot study in Japan was conducted in 2018, using the same definitions of geriatric variables in the NSQIP Geriatric Surgery Pilot Project [13].

In the current study, we compared geriatric‐associated preoperative variables and outcomes in gastroenterological surgeries between two countries. Our aim was to understand international trends in geriatric patients undergoing major gastroenterological surgery for malignancy to target care improvement for these patients in both countries.

## 2 Methods

This study was approved by the ethical committee of the Fukushima Medical University (No. 29245) and was supported with a research grant from the Ministry of Health, Labor, and Welfare in Japan (grant number 201908009A and 201908009B).

### 2.1 Patients and Data Collection

Data from the NSQIP Geriatric Surgery Pilot Project from January 2014 to June 2017 from 21 hospitals were included [12]. The NSQIP Geriatric Surgery Pilot Project included a wide variety of operative procedures (Orthopedics, General surgery, Peripheral vascular surgery, Urology, Neurosurgery, Thoracic, Plastic and Other surgeries). The Japanese geriatric surgery pilot study was conducted in 2018 across 21 hospitals. Data from the Japanese geriatric surgery pilot study were obtained from January through December 2018 [13]. The Japanese geriatric surgery pilot study targeted eight major gastroenterological surgeries (esophagectomy (Eso), total gastrectomy (TG), distal gastrectomy (DG), right hemicolectomy (RH), low anterior resection of the rectum (LAR), hepatectomy (HEP), pancreaticoduodenectomy (PD), and operation for acute diffuse peritonitis) for data collection. Geriatric‐associated variables in the Japanese study were defined and categorized in the same method as NSQIP and implemented into the National Clinical Database (NCD) system.

### 2.2 Preoperative Standard Variables and Postoperative Morbidities

Preoperative standard variables were commonly defined in the two databases: American Society of Anesthesiologists—physical status (ASA‐PS), body mass index (BMI), preoperative functional dependency, and preoperative comorbidities (diabetes mellitus (DM), dyspnea, ventilator dependency, pneumonia, chronic obstructive pulmonary disease (COPD), ascites, hypertension, acute renal failure, dialysis, disseminated cancer, open wound, steroid use for chronic condition, > 10% weight loss, bleeding disorder, transfusion, sepsis within 48 h, emergency case, smoking within 1 year, and congestive heart failure).

Postoperative 30‐day morbidities [14] were also commonly defined in both databases and included surgical site infection, wound dehiscence, pneumonia, unplanned intubation, pulmonary embolism, ventilator > 48 h, progressive renal failure, urinary tract infection, central nervous system event, cardiac arrest, myocardial infarction, deep vein thrombosis, sepsis, transfusion, coma > 24 h, peripheral nerve disorder, return to operating room, and death.

### 2.3 Geriatric‐Associated Preoperative Variables and Geriatric‐Associated Outcomes

Geriatric‐associated preoperative variables and outcomes were defined [12, 15] and characterized into four geriatric domains [12, 16]: (1) mobility (preoperative use of mobility aid, fall history within 1 year, pressure ulcer, high fall risk on discharge, and new use of mobility aid at the time of discharge), (2) function (preoperative/postoperative functional dependency, origin status, and destination after discharge), (3) cognition (preoperative history of dementia, surrogate‐signed consent, and postoperative delirium), and (4) decision‐making (advanced care planning and new DNR order).

Use of mobility aid, fall history, origin status, history of dementia, surrogate‐signed consent, and advanced care planning were preoperatively evaluated, while the remaining variables (Pressure ulcer, high fall risk, new use of mobility aid, functional dependency, destination after discharge, delirium, and new DNR order) were evaluated either on discharge or 30 days after the surgery. Origin status before hospitalization and destination after discharge were classified into four categories (living alone at home, living at home with others, not home, and unknown).

### 2.4 Statistical Analysis

Patients included in this study (1) underwent any of seven major gastroenterological surgeries (Eso, TG, DG, RH, LAR, HEP, and PD), (2) for malignant neoplasms, (3) and were aged 65 or older in each database. The seven major surgical procedures were identified using NCD code or CPT code. These codes are listed on Table S1. Patient age was categorized into three groups (65–74, 75–84, and ≥ 85). Data from each geriatric cohort in NCD and NSQIP were analyzed separately by age category. Descriptive statistics were compared. Statistical analyses were performed using Excel 2016 (Microsoft).

## 3 Results

A total of 2703 patients in NCD and 1342 patients in NSQIP were included in the present study. The number of cases for each surgical procedure is shown in Table 1. Procedures of the upper gastrointestinal tract (NCD vs. NSQIP, Eso: 12.6% vs. 2.1%, DG: 28.3% vs. 1.9%, and TG: 10.6% vs. 0.4%) were more frequent in NCD than those in NSQIP, whereas those of the lower gastrointestinal tract were more frequent in NSQIP (RH: 13.4% vs. 50.5%, and LAR: 14.0% vs. 25.9%). The frequencies of hepatectomy (NCD 8.9% vs. NSQIP 7.6%) and pancreatoduodenectomy (NCD 12.2% vs. NSQIP 11.6%) were similar between the two databases.

**TABLE 1:** Case number of each procedure.

	NCD		NSQIP	
	*n*	%	*n*	%
Esophagectomy	343	12.6	28	2.1
Distal gastrectomy	772	28.3	26	1.9
Total gastrectomy	288	10.6	5	0.4
Hepatectomy	242	8.9	102	7.6
Pancreatoduodenectomy	334	12.2	156	11.6
Right hemicolectomy	366	13.4	678	50.5
Low anterior resection	382	14.0	347	25.9
Total	2727		1342	

*Note:* Some patients in NCD underwent more than one procedure. Therefore, the total number of procedures exceeded the total number of cases.

Abbreviations: NCD, National Clinical Database; NSQIP, National Surgical Quality Improvement Program.

### 3.1 Preoperative Standard Variables

The prevalence of each preoperative standard variable by age category is summarized in Table 2. In both countries, partial and total functional dependencies were more frequent at older ages. The prevalence of dyspnea, hypertension, bleeding disorder, emergency case, and congestive heart failure increased with age in both databases. Steroid use for chronic condition and sepsis within 48 h was also more frequent in the older age group in NSQIP, although this trend was not observed in NCD. In contrast, DM and smoking decreased with age in both the NCD and NSQIP.

**TABLE 2:** Preoperative standard variables.

	NCD			NSQIP		
	65–74	75–84	≥ 85	65–74	75–84	≥ 85
	*n* = 1481	*n* = 1061	*n* = 161	*n* = 743	*n* = 460	*n* = 139
ASA‐PS						
ASA 1	15.5%	8.2%	6.8%	0.4%	0.0%	0.0%
ASA 2	75.2%	73.0%	60.2%	29.0%	15.6%	7.2%
ASA 3	9.0%	18.7%	31.7%	64.3%	74.8%	71.2%
ASA 4	0.1%	0.1%	1.2%	6.3%	9.6%	20.9%
ASA 5	0.2%	0.0%	0.0%	0.0%	0.0%	0.7%
BMI						
Underweight (< 18.5)	13.5%	10.7%	18.0%	2.0%	3.0%	6.5%
BMI 18.5–24	65.0%	69.1%	68.9%	26.6%	35.2%	37.4%
BMI 25–29	18.7%	18.5%	11.8%	35.7%	37.0%	35.3%
BMI 30–34	2.2%	1.2%	0.6%	21.7%	16.7%	16.5%
BMI 35–39	0.1%	0.2%	0.0%	7.9%	5.7%	2.9%
BMI 40 and over	0.5%	0.3%	0.6%	6.1%	2.4%	1.4%
Functional dependency						
Partially	1.4%	5.3%	14.3%	2.2%	4.6%	13.0%
Totally	0.1%	0.7%	4.3%	0.4%	0.6%	1.4%
DM	22.2%	23.2%	18.0%	22.3%	21.3%	16.6%
Dyspnea	0.6%	1.6%	1.9%	7.1%	11.1%	15.8%
Ventilator dependent	0.1%	0.0%	0.0%	0.5%	0.0%	0.0%
Pneumonia on surgery	0.3%	0.7%	1.2%	0.3%	0.2%	2.9%
COPD	5.5%	8.8%	5.0%	6.5%	8.7%	7.2%
Ascites	0.7%	0.4%	3.1%	0.8%	0.2%	0.0%
Hypertension	44.2%	52.6%	52.2%	61.0%	70.9%	79.1%
Acute renal failure	0.0%	0.0%	0.0%	0.3%	0.2%	0.7%
Dialysis	0.7%	0.9%	1.2%	0.3%	0.9%	1.4%
Disseminated cancer	1.2%	1.3%	1.2%	11.0%	8.3%	5.0%
Open wound	0.0%	0.0%	0.6%	2.8%	2.6%	5.8%
Steroid use for chronic condition	1.7%	1.3%	0.6%	4.7%	6.5%	10.4%
> 10% weight loss	3.6%	5.2%	4.3%	5.7%	6.5%	6.5%
Bleeding disorder	2.8%	5.7%	8.7%	4.2%	7.0%	13.7%
Transfusion	0.5%	1.3%	3.1%	1.8%	4.1%	2.2%
Sepsis within 48 h	0.3%	0.1%	0.6%	4.7%	7.2%	13.0%
Emergency case	0.7%	0.8%	1.2%	5.5%	7.6%	11.5%
Smoking within 1 year	24.6%	15.0%	7.5%	14.9%	7.8%	1.4%
Congestive heart failure	0.4%	0.2%	4.3%	1.4%	2.0%	2.2%

Abbreviations: ASA‐PS, American Society of Anesthesiologists—physical status; BMI, body mass index; COPD, chronic obstructive pulmonary disease; DM, diabetes mellitus; NCD, National Clinical Database; NSQIP, National Surgical Quality Improvement Program.

The prevalence of preoperative functional dependency, DM, ventilator dependency, pneumonia, COPD, acute renal failure, and dialysis in each age category was similar between the two countries. On the other hand, higher ASA scores and BMI were observed in NSQIP patients. Patients with ASA ≥ 3 were 77.6% in NSQIP and 14.4% in NCD. Patients with BMI ≥ 30 were 30.4% in NSQIP and 2.3% in NCD. Moreover, dyspnea, hypertension, disseminated cancer, open wound, steroid use for chronic conditions, bleeding disorders, sepsis within 48 h (4.7%–13.0% in NSQIP vs. 0.1%–0.6% in NCD), and emergency cases (5.5%–11.5% in NSQIP vs. 0.7%–1.2% in NCD) were more frequent in NSQIP than in NCD. By contrast, smoking patients were more frequent in NCD. In addition, the prevalence of patients with ascites and congestive heart failure in patients ≥ 85 was higher in NCD than in NSQIP.

### 3.2 Postoperative Morbidities

The frequencies of postoperative morbidities at every age category are summarized in Table 3. The incidence of ventilation > 48 h and sepsis was elevated in patients ≥ 85 in both databases. In NSQIP, myocardial infarction, deep vein thrombosis, and transfusion slightly increased with age. The other morbidities did not seem to be associated with age in both cohorts.

**TABLE 3:** Postoperative morbidities.

	NCD			NSQIP		
	65–74	75–84	≥ 85	65–74	75–84	≥ 85
	*n* = 1481	*n* = 1061	*n* = 161	*n* = 743	*n* = 460	*n* = 139
SSI						
Superficial	3.5%	4.5%	3.7%	3.1%	3.5%	2.2%
Deep incisional	1.4%	0.8%	0.0%	0.5%	0.4%	0.0%
Organ/Space	3.2%	1.8%	0.0%	6.3%	6.3%	5.8%
Wound dehiscence	0.3%	0.6%	0.0%	0.5%	0.2%	0.7%
Pneumonia	5.3%	5.0%	6.2%	3.1%	3.9%	4.3%
Unplanned intubation	1.4%	1.5%	0.6%	3.4%	3.9%	3.6%
Pulmonary embolism	0.3%	0.4%	0.0%	0.5%	1.3%	0.7%
Ventilator > 48 h	1.7%	1.3%	2.5%	3.0%	2.8%	5.0%
Progressive renal failure	1.3%	1.6%	0.6%	0.8%	1.3%	1.4%
UTI	0.7%	1.9%	0.6%	1.2%	2.8%	2.9%
CNS event (stroke)	0.1%	0.6%	0.0%	0.4%	0.0%	1.4%
Cardiac arrest	0.3%	0.2%	0.0%	1.2%	0.9%	0.0%
MI	0.1%	0.1%	0.0%	0.7%	1.3%	2.9%
DVT	1.1%	0.8%	0.6%	1.6%	2.0%	2.9%
Sepsis	1.6%	1.2%	2.5%	7.1%	8.5%	11.5%
Transfusion	4.5%	4.4%	2.5%	11.3%	15.2%	18.7%
Coma > 24 h	0.3%	0.2%	0.6%	0.0%	0.0%	0.0%
Peripheral nerve disorder	0.2%	0.2%	0.0%	0.0%	0.0%	0.0%
Return to OR	4.0%	3.0%	0.6%	5.0%	4.4%	4.3%
Thirty‐day mortality	0.3%	0.2%	0.0%	2.1%	2.4%	5.0%

Abbreviations: CNS, central nervous system; DVT, deep vein thrombosis; MI, myocardial infarction; NCD, National Clinical Database; NSQIP, National Surgical Quality Improvement Program; OR, operating room; SSI, surgical site infection; UTI, urinary tract infection.

Surgical site infection (organ/space), unplanned intubation, pulmonary embolism, ventilator > 48 h, urinary tract infection, myocardial infarction, deep vein thrombosis, sepsis (7.1%–11.5% in NSQIP vs. 1.2%–2.5% in NCD), and transfusion were more frequent in NSQIP than in NCD. Surgical site infection (superficial) and pneumonia at all age categories were more frequently observed in NCD than in NSQIP.

### 3.3 Geriatric‐Associated Preoperative Variables

**FIGURE 1:**
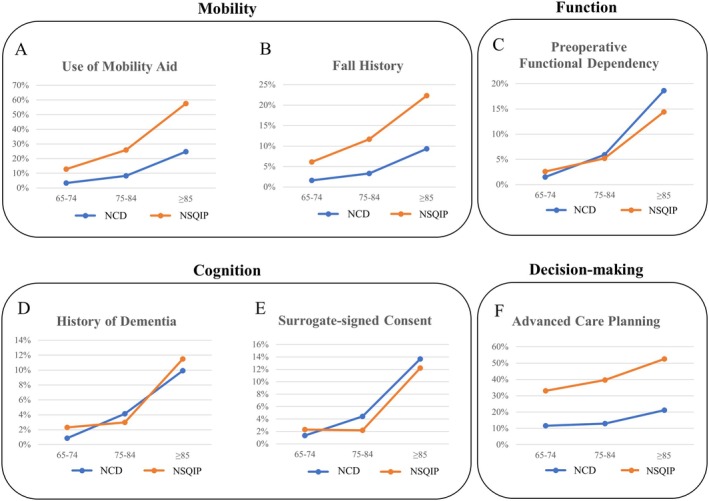
Geriatric‐associated preoperative variables of two databases represented by age category. Preoperative functional dependency included partial and total dependent patients. All variables increased with age. Preoperative functional dependency (C), history of dementia (D), and surrogate‐signed consent (E) were similar between two databases, whereas the use of mobility aid (A), fall history (B), and advance care planning (F) were more frequent in NSQIP than in National Clinical Database (NCD).

Use of mobility aid, fall history, history of dementia, surrogate‐signed consent, and advanced care planning increased with advanced age in both databases (Table 4, Figure 1A,B,D–F).

**TABLE 4:** Geriatric‐associated preoperative variables.

Geriatric domain	Variable	NCD			NSQIP		
		65–74	75–84	≥ 85	65–74	75–84	≥ 85
		*n* = 1481	*n* = 1061	*n* = 161	*n* = 743	*n* = 460	*n* = 139
Mobility	Use of mobility aid	3.3%	8.3%	24.8%	12.8%	25.9%	57.6%
	Fall history	1.6%	3.3%	9.3%	6.1%	11.7%	22.3%
Function	Origin status						
	Live alone at home	11.3%	11.3%	11.2%	20.2%	29.3%	38.1%
	Live at home with others	83.5%	82.3%	71.4%	78.9%	68.7%	59.0%
	Not home	4.6%	5.9%	15.5%	0.9%	2.0%	2.9%
Cognition	History of dementia	0.9%	4.1%	9.9%	2.3%	3.0%	11.5%
	Surrogate‐signed consent	1.4%	4.4%	13.7%	2.3%	2.2%	12.2%
Decision‐making	Advanced care planning	11.6%	13.0%	21.1%	33.0%	39.6%	52.5%

Abbreviations: NCD, National Clinical Database; NSQIP, National Surgical Quality Improvement Program.

The history of dementia and surrogate‐signed consent was similar between the two cohorts (Table 4, Figure 1D,E). Approximately 10% of patients with age ≥ 85 had dementia before hospitalization in both databases. Similarly, informed consent for surgery was obtained from a surrogate in more than 10% of patients in this age category. Meanwhile, the frequency of mobility aid, fall history, and advanced care planning in NSQIP was more than twice as high as those in NCD in all age categories (Figure 1A,B,F). Patients whose origin was not home increased with age in NCD and NSQIP, though with larger overall rates in NCD (age ≥ 85: 2.9% in NSQIP vs. 15.5% in NCD). Patients living alone at home were more frequent overall in NSQIP and increased with advancing age (age ≥ 85: 38.1% in NSQIP vs. 11.2% in NCD).

### 3.4 Geriatric‐Associated Outcomes

**FIGURE 2:**
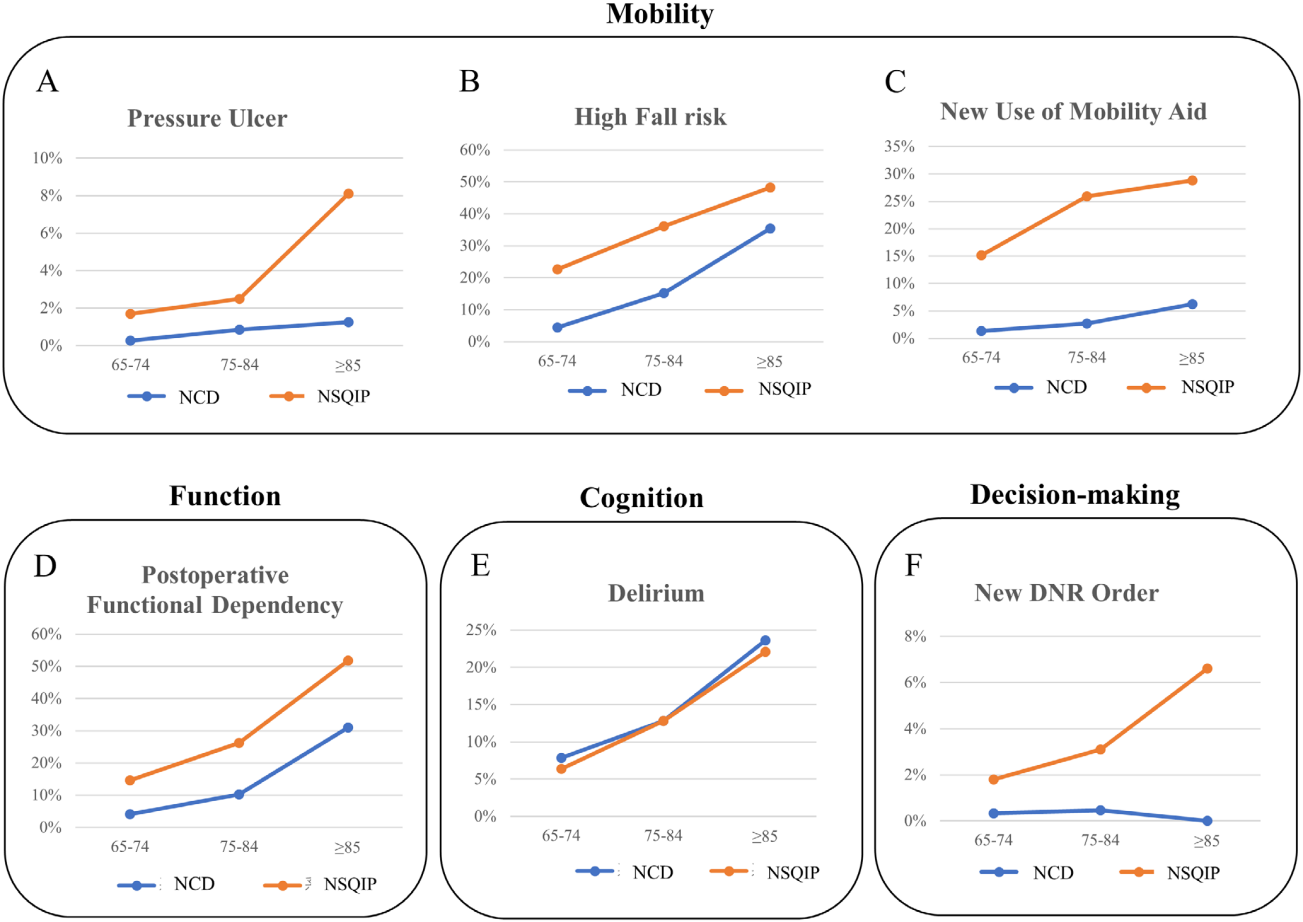
Relationships of geriatric associated outcomes with age. Geriatric associated outcomes of two databases are represented by age category. Most outcomes increased in frequency as patients became older. Pressure ulcer (A), high fall risk (B), new use of mobility aid (C), postoperative functional dependency (D), and new DNR order (F), were more frequent in NSQIP than in National Clinical Database (NCD), whereas the prevalence of delirium (E), was similar between two databases.

Table 5 demonstrates the relationship between geriatric‐associated outcomes and age categories, with further visualization in Figure 2. Pressure ulcer, high fall risk on discharge, new use of mobility aid, postoperative functional dependency, and delirium increased with age in both databases.

**TABLE 5:** Geriatric‐associated outcomes.

Geriatric domain	Outcome	NCD			NSQIP		
		65–74	75–84	≥ 85	65–74	75–84	≥ 85
		*n* = 1481	*n* = 1061	*n* = 161	*n* = 743	*n* = 460	*n* = 139
Mobility	Pressure ulcer	0.3%	0.8%	1.2%	1.7%	2.5%	8.1%
	High fall risk	4.5%	15.2%	35.4%	22.6%	36.1%	48.2%
	New use of mobility aid	1.4%	2.7%	6.2%	15.2%	25.9%	28.8%
Function	Functional dependency						
	Partially	3.8%	9.1%	24.8%	13.9%	24.3%	48.9%
	Totally	0.2%	1.1%	6.2%	0.8%	2.0%	2.9%
	Destination after discharge						
	Home alone	51.2%	42.9%	36.0%	11.9%	13.9%	7.2%
	Home with others	45.2%	48.6%	41.0%	75.2%	61.3%	43.9%
	Not home	3.5%	8.5%	23.0%	9.7%	21.1%	42.4%
Cognition	Delirium	7.8%	12.8%	23.6%	6.4%	12.8%	22.1%
Decision‐making	New DNR order	0.3%	0.5%	0.0%	1.8%	3.1%	6.6%

Abbreviations: NCD, National Clinical Database; NSQIP, National Surgical Quality Improvement Program.

Comparing geriatric‐associated outcomes between two databases, the prevalence of delirium was almost identical between two cohorts (Figure 2E). More than 20% of patients age 85 or older developed delirium after surgery in both databases. Pressure ulcer, high fall risk, new use of mobility aid, and postoperative functional dependency were more frequently observed in NSQIP than in NCD (Figure 2A–D). Patients living at home alone after discharge were more frequent in NCD, whereas patients discharged to non‐home facilities were higher in NSQIP. The incidence of new DNR orders was also higher in NSQIP geriatric patients.

**FIGURE 3:**
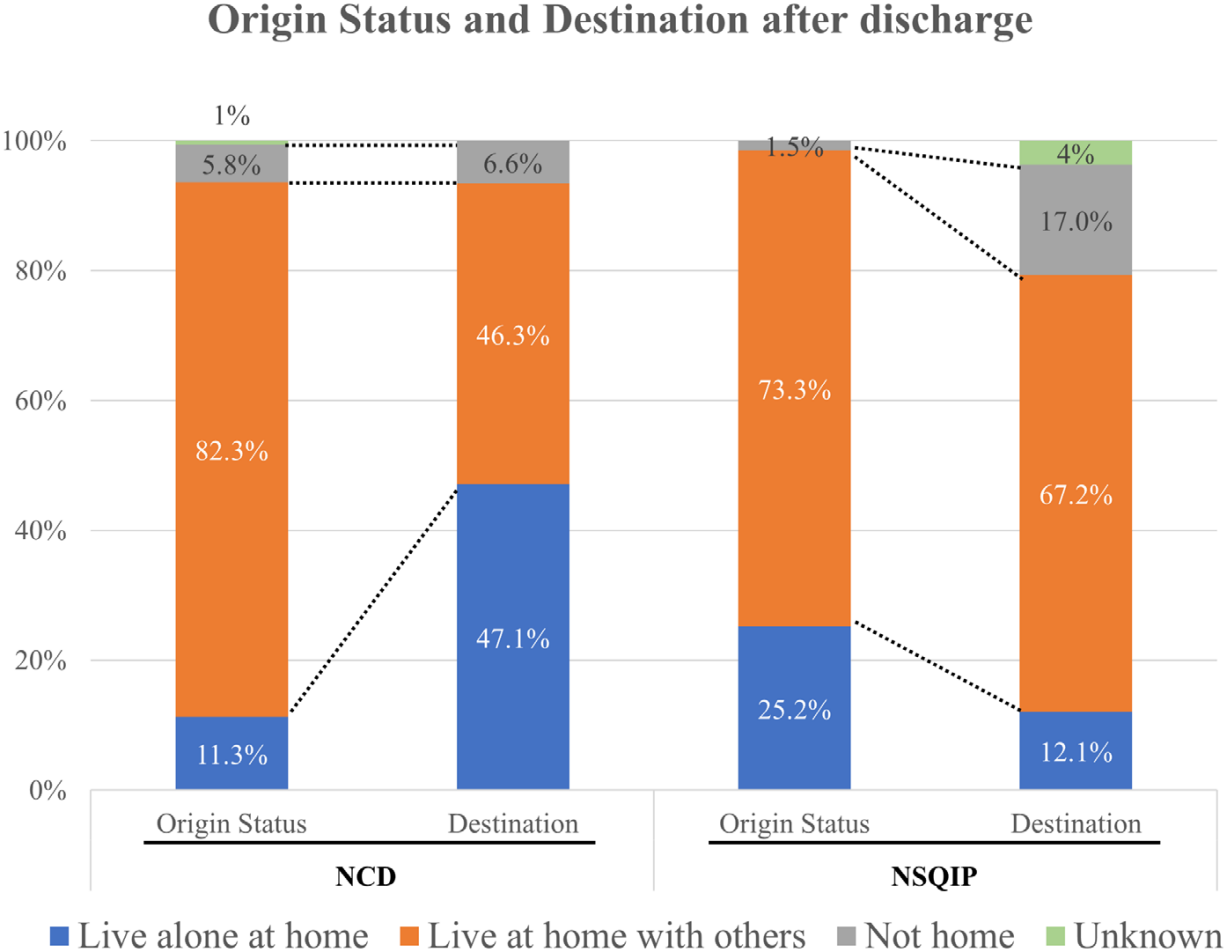
Origin status and destination after discharge of all geriatric cases in National Clinical Database (NCD) or NSQIP. Postoperatively, patients living alone at home and those with non‐home destinations increased in NCD and NSQIP, respectively.

Figure 3 demonstrates origin status and destination after discharge of all cases in NCD or NSQIP across all ages 65 or greater. Origin status before hospitalization was mostly “home” in both countries. The proportion of patients preoperatively living at home with others was slightly higher in NCD than in NSQIP. However, Japanese patients living at home with others plunged postoperatively, and patients living alone at home postoperatively were more than four times greater (origin vs. destination: 11.3% vs. 47.1%). Patients living at a location other than home increased postoperatively in NSQIP (origin vs. destination: 1.5% vs. 17.0%), and patients living alone at home after discharge decreased (origin vs. destination: 25.2% vs. 12.1%). The proportion of patients living in a non‐home location was relatively constant before and after surgery in NCD (origin vs. destination: 5.8% vs. 6.6%).

## 4 Discussion

In the present study, geriatric‐associated preoperative risk factors and surgical outcomes were compared between patients undergoing major gastroenterological surgery for malignancy in Japan and the US. To our knowledge, this is the first study to compare these risk factors and outcomes between these two countries, which may provide further context for targeting surgical quality improvement for geriatric surgical patients.

Relationships between standard preoperative variables and age were similar in both databases. Dyspnea, hypertension, bleeding disorder, emergency case, and congestive heart failure increased with patient age. This is consistent with the known natural progression of disease in aging patients, and the surgical triage for emergency cases in this population. In contrast, DM and smoking decreased with advancing age. The trend in DM prevalence at advanced age is contrary to recent data suggesting increased risk in older adults [17, 18]. This trend may not be readily observed in our study population due to selection effects for surgical patients with malignancy. Smoking decline among older adults has been demonstrated in recent data from both countries [19, 20]. Remaining comorbidities showed little observable relationship with age.

The frequency of some preoperative standard variables differed between Japan and the US. BMI was higher across all age groups in NSQIP. This is consistent with prior work reporting higher obesity rates in NSQIP than NCD (BMI ≥ 30, NSQIP vs. NCD: 25.8%–33.3% vs. 1.5%–2.5%) [21]. Moreover, the frequencies of dyspnea, hypertension, disseminated cancer, open wound, steroid use for chronic conditions, bleeding disorder, sepsis within 48 h, and emergency cases were more frequent in the NSQIP than in NCD. Ascites, preoperative transfusion, and congestive heart failure in patients age ≥ 85, and smoking at all age categories were slightly more frequent in NCD. These differences may be reflected by the characteristics of each country. In this study, the ASA score was higher in NSQIP than NCD. This is consistent with prior work demonstrating differences in ASA scores between NSQIP and the NCD [21], though it may also be due to disagreement in ASA classification in the NCD. However, prior work has reported higher disagreement in ASA classification at lower levels (classes 1 and 2) than more clinically significant levels (classes 3 and 4) [22].

In contrast with preoperative standard variables, most postoperative morbidities demonstrated little variation by age stratification, except for ventilator > 48 h and sepsis, which were elevated in patients ≥ 85 in both databases. The relationship between age and postoperative morbidities has been reported. Lee et al. [5] have investigated gastrectomy outcomes between patients ≥ 75 and those aged 65–74, reporting no difference in postoperative morbidities between the two groups despite elderly patients having more comorbidities. Regarding pancreaticoduodenectomy, Zhang et al. [23] have reported no significant differences in overall morbidity and surgical complications between patients ≥ 70 and patients < 70 years old, while Mizuma et al. [24] have reported that older age was a risk factor for severe complications. Zawadzki et al. [25] have evaluated colorectal cancer in the elderly population, reporting higher postoperative complications in patients ≥ 75 years old. The association between age and postoperative morbidity in liver resection has been controversial [6, 7]. While these previous studies were procedure‐targeted, our study includes outcomes from seven major gastroenterological procedures with added age discrimination of 85 years and older. This may allow more precise determination of age‐related differences in postoperative morbidity while sacrificing discrimination by surgical procedure.

The incidence of several postoperative morbidities (surgical site infection, pneumonia, unplanned intubation, pulmonary embolism, ventilator > 48 h, urinary tract infection, myocardial infarction, deep vein thrombosis, sepsis, and transfusion) varied between two databases. Postoperative sepsis in NSQIP was four times more frequent than in NCD in each age category. Moore et al. [26] demonstrate that emergency surgery increases postoperative sepsis in general surgery. In this study, emergency cases were about 0.8% of all cases in NCD, and 6.9% of all cases in NSQIP. The increased rate of emergency cases in NSQIP might contribute to the greater observed incidence of sepsis. Other preoperative risk factors elevated in NSQIP versus NCD, such as preoperative sepsis within 48 h and deep space SSI, may have contributed to this increased rate of postoperative sepsis [27].

The directional relationship between advancing age and geriatric‐associated preoperative risk factors and outcomes was similar in both countries (Tables 4, 5). However, with respect to geriatric domains, frequencies differed between the two countries. Perioperative elements of cognition were similar between the two databases. A higher frequency of risk factors and adverse outcomes within the mobility and function domain was observed in NSQIP.

The NSQIP cohort had increased frequencies of preoperative mobility aid use and fall history across all age groups compared to NCD (Table 4). New use of mobility aid on discharge was largely increased in patients aged ≥ 85 in comparison with those aged 65–74 in both databases (NSQIP +13% increase, NCD +5% increase). This is consistent with prior work demonstrating an increased need for assistance with advanced age [16, 28, 29]. Preoperative functional dependencies were comparable across age categories in both databases. Functional dependency on discharge was higher in NSQIP than NCD, with partial and total functional dependency exceeding 50% among patients aged ≥ 85 in NSQIP. This may be due to differing discharge practices between the US and Japan, where discharge permission is not granted in Japan until functional dependency is restored.

Differences in average hospital stay between Japan and the US have been demonstrated, which may be attributed to differences in health care systems [30]. Previous reports showed that the length of hospital stay of Eso, HEP, PD, RH, and LAR was shorter in NSQIP than that in NCD (Eso: 10 vs. 41.7 days, HEP: 8 vs. 22 days, PD: 9 vs. 31 days, RH: 5 vs. 14 days, LAR: 6 vs. 16 days) [21, 31, 32, 33, 34]. Therefore, the assessment time points of postoperative functional dependency, high fall risk on discharge, and new use of mobility aid were much earlier in the postoperative course in NSQIP than in NCD. This may affect observed postoperative function and mobility.

The majority of patients were admitted to the hospital from home in both countries. Most of them lived with others at home. However, destinations after discharge differed between both countries. In Japan, home status was relatively constant preoperatively and postoperatively. Although 98% of patients in NSQIP were admitted from home, only 79% were discharged home, representing an increased need for assistance subsequently absorbed by nursing facilities. Increased utilization and improvement of postoperative care facilities may represent an opportunity to minimize hospital length of stay for geriatric patients in Japan. Regarding discharge destinations in Japan, the proportion of patients discharged to live alone at home increased, whereas the proportion of patients discharged to live at home with others decreased. This unexpected result could not be explained currently, and a survey for further investigation is imperative.

Patients with advanced care planning were fewer in NCD than in NSQIP, where nearly 40% of all included patients had some form documented. Advanced care planning is demonstrably less practiced in Japan compared with the US [35], indicating another consideration for improving the Japanese health care system for older adults.

One limitation of this study is that the proportions of surgical procedures were different between both databases. Furthermore, information on whether surgery was minimally invasive was lacking. These factors may have affected the study results. However, the primary goal of this study was to characterize perioperative geriatric characteristics across major gastroenterological surgeries between two countries. Given the scope of this comparison at the population level and the goal to inform far‐reaching future improvement efforts, adjustment was not conducted for surgical procedure type. The second limitation is the variability in patient backgrounds. Eliminating the influence of background factors on the outcomes would result in a more accurate comparison and results. However, the data collected from both countries for this study represented population‐level rather than individual patient data, making adjustments difficult. In addition, in compliance with the American Statistical Association statement on *p*‐values and statistical significance, when analyzing a large number of variables, statistical tests for differences in background factor frequencies or outcome incidences were not conducted without a prespecified hypothesis. Consequently, no statistical comparison of background factors between the two countries was performed in this study. A third limitation is the variability in assessment time points on discharge, because the length of hospital stay tends to differ between these two countries. Another method to evaluate these postoperative characteristics, accounting for differences in inpatient postoperative periods would be to compare functional dependency and physical function at 30 days postoperatively. However, because hospital stay was shorter in NSQIP and the data on postoperative 30 days were missing in a substantial portion of NSQIP patients, this comparison was not performed (Table S2). These databases consist of nationwide multicenter voluntary participation in each country and may have limited generalizability to nonparticipating centers. Nevertheless, we anticipate participating centers to have a vested interest in geriatric surgical patients, which may lead to an underestimation of complications and comorbidity in the geriatric surgical population at large. These limitations may be ameliorated by prospective studies that align surgical procedures and patient characteristics, nationwide data sampling, and robust data collection of postoperative 30‐day functional outcomes.

## 5 Conclusions

Despite differences in demographic features and preoperative comorbidities, geriatric‐associated preoperative risk factors and outcomes were associated with advancing age in two countries. Although elements of perioperative cognition were similar between the two countries, the elements of mobility and function were more deteriorated in NSQIP than in NCD. Further investigation is warranted to continue to inform improvement in both participating countries.

## Author Contributions

**Yasuhide Kofunato:** investigation, visualization, writing – original draft. **Xane Peters:** writing – review and editing. **Arata Takahashi:** formal analysis, investigation, methodology, resources, writing – review and editing. **Mark E. Cohen:** formal analysis, methodology, resources. **Hiraku Kumamaru:** formal analysis, methodology, resources, writing – review and editing. **Mitsukazu Gotoh:** conceptualization, methodology, project administration, supervision, writing – review and editing. **Yoshihiro Kakeji:** conceptualization, project administration, supervision, writing – review and editing. **Yasuyuki Seto:** conceptualization, project administration, supervision, writing – review and editing. **Yuko Kitagawa:** supervision, writing – review and editing. **Ken Shirabe:** supervision, writing – review and editing. **Hideki Ueno:** supervision, writing – review and editing. **Hiroaki Miyata:** conceptualization, project administration, supervision, writing – review and editing. **Clifford Y. Ko:** conceptualization, methodology, project administration, supervision, writing – review and editing. **Shigeru Marubashi:** conceptualization, funding acquisition, investigation, project administration, supervision, writing – review and editing.

## Ethics Statement

The protocol for this research project has been approved by a suitably constituted Ethics Committee of the institution and it conforms to the provisions of the Declaration of Helsinki. Committee of the Fukushima Medical University, Approval No. 29245. This study is an observational study based on data registered in databases established in both countries, and informed consent is in accordance with the conventions of each database. Registry of this study was not applicable.

## Conflicts of Interest

Author Yuko Kitagawa is Editor‐in‐Chief of Annals of Gastroenterological Surgery. Author Hideki Ueno is Chief Associate Editor of Annals of Gastroenterological Surgery. Authors Yoshihiro Kakeji and Ken Shirabe are editorial board members of Annals of Gastroenterological Surgery.

## Supporting information


Data S1.
